# US medical and surgical society position statements on physician-assisted suicide and euthanasia: a review

**DOI:** 10.1186/s12910-020-00556-5

**Published:** 2020-11-03

**Authors:** Joseph G. Barsness, Casey R. Regnier, C. Christopher Hook, Paul S. Mueller

**Affiliations:** 1 Division of Hematology, Mayo Clinic, Rochester, MN USA; 2 Department of Internal Medicine, Mayo Clinic Health System–Franciscan Healthcare in La Crosse, 800 West Ave S, La Crosse, WI 54601 USA; 3 Hamline University, St. Paul, MN USA; 4 Augsburg University, Minneapolis, MN USA

**Keywords:** Assisted death, Euthanasia, PAD, PAS, Physician-assisted suicide

## Abstract

**Background:**

An analysis of the position statements of secular US medical and surgical professional societies on physician-assisted suicide (PAS) and euthanasia have not been published recently. Available statements were evaluated for position, content, and sentiment.

**Methods:**

In order to create a comprehensive list of secular medical and surgical societies, the results of a systematic search using Google were cross-referenced with a list of societies that have a seat on the American Medical Association House of Delegates. Societies with position statements were identified. These statements were divided into 5 categories: opposed to PAS and/or euthanasia, studied neutrality, supportive, acknowledgement without statement, and no statement. Linguistic analysis was performed using RapidMinder in order to determine word frequency and sentiment respective to individual statements. To ensure accuracy, only statements with word counts > 100 were analyzed. A 2-tailed independent *t* test was used to test for variance among sentiment scores of opposing and studied neutrality statements.

**Results:**

Of 150 societies, only 12 (8%) have position statements on PAS and euthanasia: 11 for PAS (5 opposing and 4 studied neutrality) and 9 for euthanasia (6 opposing and 2 studied neutrality). Although the most popular words used in opposing and studied neutrality statements are similar, notable exceptions exist (*suicide*, *medicine*, and *treatment* appear frequently in opposing statements, but not in studied neutrality statements, whereas *psychologists*, *law*, and *individuals* appear frequently in studied neutrality statements, but not in opposing statements). Sentiment scores for opposing and studied neutrality statements do not differ (mean, 0.094 vs. 0.104; *P* = 0.90).

**Conclusions:**

Few US medical and surgical societies have position statements on PAS and euthanasia. Among them, opposing and studied neutrality statements share similar linguistic sentiment. Opposing and studied neutrality statements have clear differences, but share recommendations. Both opposing and studied neutrality statements cite potential risks of PAS legalization and suggest that good palliative care might diminish a patient’s desire for PAS.

## Background

Physician-assisted suicide (PAS) and euthanasia are highly debated and controversial topics in the United States. In PAS, a patient ingests a drug prescribed by a physician for the purpose of causing the patient’s death, in order to relieve unacceptable symptoms or quality of life. Currently, California [1], Colorado [2], Hawaii [3], Maine [4], Oregon [5], Vermont [6], Washington [7], and the District of Columbia [8] have legalized PAS. (The Montana Supreme Court ruled that PAS does not conflict with Montana public policy [9]) The other US states prohibit PAS and punish it by law [10]. In euthanasia, a physician (or someone else) administers a lethal drug. Euthanasia is illegal throughout the United States [10].

Laws concerning PAS, also known as *physician-assisted death* (PAD), are generally more rigid in the United States than in countries where PAD is legal. For instance, in the Netherlands, PAS and euthanasia are legal for adults (or for persons age 12 through 17 years with parental involvement). Requests for PAD “often come from patients experiencing unbearable suffering with no prospect of improvement. Their request must be made earnestly and with full conviction… However, patients have no absolute right to euthanasia and doctors no absolute duty to perform it” [11]. Currently, no US state allows PAS for persons younger than 18 years, and PAS is illegal in the absence of a severe physical ailment that will result in natural death within 6 months [1–3, 5–8]. The ethical permissibility of PAS and euthanasia, along with considerations regarding what constitutes patient autonomy in decision-making associated with the dying process, creates an emotionally provocative and divisive debate. The position statements of medical and surgical professional societies concerning PAS and euthanasia may reflect complexities.

The statements of professional societies guide clinicians on various topics, such as disease prevention and management. For PAS and euthanasia, such statements inform, provide multiple (sometimes opposing) perspectives, and advocate for specific positions. Because PAS and euthanasia involve physician action, physicians understandably may turn to their professional societies for guidance on these topics. A comprehensive analysis of such statements issued by secular US medical and surgical professional societies has not been published recently. Therefore, in this cross-sectional study, we determined the number of secular US societies that have position statements about PAS and euthanasia and the positions they have taken. We also analyzed the contents and conducted a linguistic analysis of the statements.

## Methods

We developed a comprehensive list of secular US medical and surgical professional societies with use of 2 methods. To do so, we first conducted a systematic Google search using the phrase “American […] medical societies,” with the bracket including a name of a specialty derived from the Mayo Clinic directory. An original search was performed for each specialty in the directory, and the respective first 2 Google search pages were analyzed for societies. Second, we obtained a list of specialty societies from the American Medical Association (AMA) website [12]. This list reflects organizations entitled to a seat in the AMA House of Delegates. It served to provide a cross-reference for our Google search results. In a combination of these 2 methods, a comprehensive list was created of the US specialty-based medical and surgical professional societies.

We determined the positions of these societies on PAS or euthanasia, or both. These positions were organized into 5 categories: supportive, opposed, studied neutrality, acknowledgement without statement, and no statement. Studied neutrality position statements are characterized by an understanding of the practical concerns associated with PAS or euthanasia and the persistent desire of some patients for PAS or euthanasia despite these concerns. The American Academy of Hospice and Palliative Medicine (AAHPM) position statement [13] exemplifies a studied neutrality statement. To determine whether a society had publicly issued a position statement on PAS or euthanasia, or both, the society’s official website was consulted. If no such statement was available on the website, an email was sent to a society contact listed on the website. If the society website did not provide a statement and no official position statement was publicly available, the society was categorized as having no statement.

For each available position statement, linguistic analysis was performed using a data science software platform (RapidMiner; RapidMiner) [14]. From each statement, so-called stop words (such as *is*, *a*, or *the*) and the name of the society were filtered out because these words provided no insights into the intent of the statement authors. The other words were sorted by frequency of use, producing a chart of each statement’s top word choices. In addition, a given statement’s words that were used more than once were processed by a sentiment analysis tool (Twinword; Twinword Inc). This approach relates word choice to emotional attitude and generates a quantitative score of emotional positivity. Medhat et al. [15] provided a comprehensive survey of sentiment analysis approaches, including a description of the dictionary-based approach used in the present study. To ensure the tool’s generated score was reflective of the statement, we analyzed only the statements with more than 100 words. A mean score was generated for opposing statements and studied neutrality statements. Sentiment scores were compared among the opposing and studied neutrality statements with a 2-tailed independent *t* test.

## Results

Our search methodology identified a total of 150 distinct secular US medical and surgical professional societies (Additional file 1: Table S1). Of these, only 12 (8%) had position statements regarding PAS or euthanasia, or both (Table 1). Eleven societies (7%) had statements on PAS. Of these, 5 (45%) had positions opposing PAS; 4 (36%), positions of studied neutrality; and 2 (18%), acknowledgment of PAS without a position. No society had a statement overtly in support of PAS. Regarding euthanasia, 9 societies (6%) had position statements: 6 (67%), positions opposing euthanasia; 2 (22%), positions of studied neutrality; and 1 (11%), acknowledgement of euthanasia without a position. No society had a statement overtly supporting euthanasia. Three societies have had their position statements published in peer-reviewed journals (American Academy of Neurology [AAN] [16], American College of Obstetricians and Gynecologists [17], and American College of Physicians [ACP] [19]).

**Table 1:** Positions on PAS and Euthanasia of US medical and surgical specialty societies with position statements

Society	Position statements	
	PAS	Euthanasia
American Academy of Hospice and Palliative Medicine [13]	Studied neutrality	No statement
American Academy of Neurology [16]	Studied neutrality	Opposed
American College of Obstetricians and Gynecologists [17]	Acknowledge	Acknowledge
American College of Pediatricians [18]	No statement	Opposed
American College of Physicians [19]	Opposed	Opposed
American Medical Association [20, 21]	Opposed	Opposed
American Pharmacists Association [22]	Studied neutrality	Studied neutrality
American Psychiatric Association [23]	Acknowledge	Opposed
American Psychological Association [24]	Studied neutrality	Studied neutrality
American Society of Anesthesiologists [25]	Opposed	No statement
National Hospice and Palliative Care Organization [26]	Opposed	No statement
Society for Post-Acute and Long-term Care Medicine [27]	Opposed	Opposed

*PAS* physician-assisted suicide

**Fig. 1:**
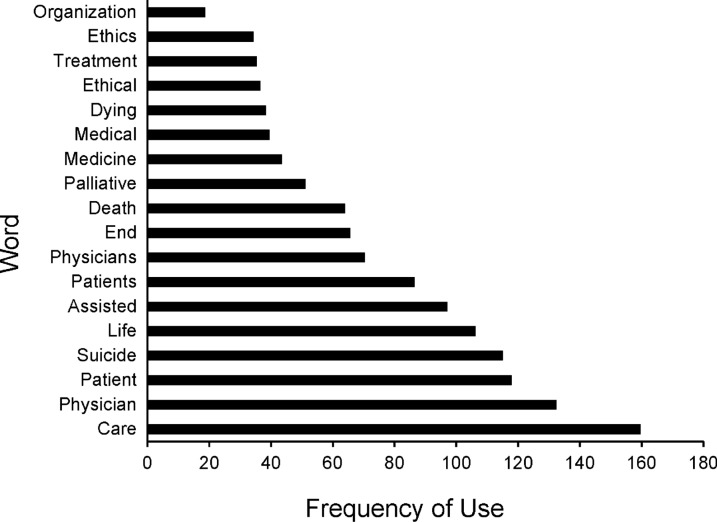
Most popular words in opposing position statements among 150 US medical and surgical professional societies

**Fig. 2:**
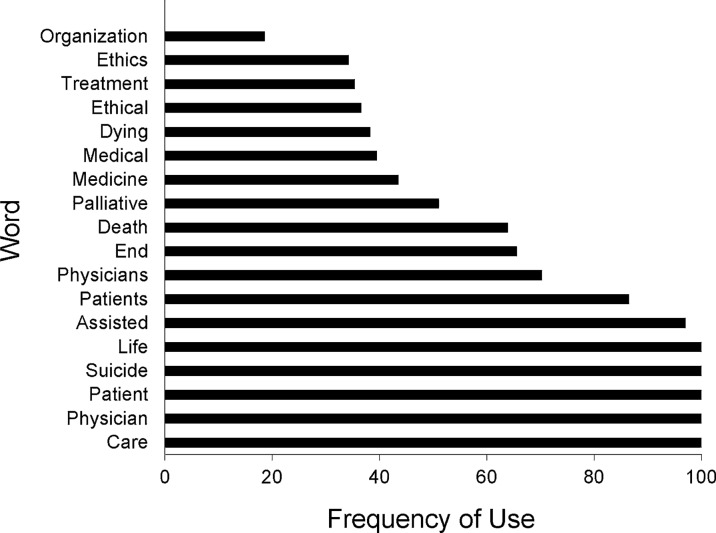
Most popular words in studied neutrality position statements among 150 US medical and surgical professional societies

The most popular words used in opposing position statements and studied neutrality statements are shown in Figs. 1 and 2. *Suicide*, *medicine*, and *treatment* appear frequently in opposing statements but not in studied neutrality statements. By comparison, *psychologists*, *law*, and *individuals* appear frequently in studied neutrality statements but not in opposing statements. Otherwise, the words used in these 2 statement types are similar. In linguistic analysis, the mean sentiment score was 0.094 for opposing statements and 0.104 for studied neutrality statements, a nonsignificant difference (*P* = 0.90). Of note, Twinword categorizes any sentiment score below − 0.05 as negative and any score above 0.05 as positive [28]. Thus, given the mean scores in this study, the opposing and studied neutrality statements both used emotionally positive language.

From a content standpoint, the opposing statements generally argue that PAS is problematic in practice and in society. The statements of the Society for Post-Acute and Long-term Care Medicine (AMDA), ACP, AMA, and the American Society of Anesthesiologists (ASA) assert that PAS contradicts the healing role of the physician [19, 20, 25, 27]. To counter the claim that PAS is a legitimate approach to symptom control in extreme cases, the statements of AMDA, ACP, and the National Hospice and Palliative Care Organization (NHPCO) highlight the legal permissibility of palliative sedation. Finally, ACP and NHPCO mention that the US Supreme Court has ruled that no legal right to PAS exists. Studied neutrality statements provide supportive and cautionary arguments concerning PAS. The statements of AAHPM, AAN, and the American Pharmacists Association acknowledge the complexities of the physician–patient relationship [13, 16, 22], and American Psychological Association (APA) acknowledges the social complexities of the medical environment [24]. Each of these statements emphasizes patient autonomy in decision-making associated with the dying process.

The studied neutrality statements generally support further study of palliative care techniques or physician ethics training, or a combination. Opposing statements (ACP [19] and AMA [20]) and studied neutrality statements (AAHPM [13] and AAN [16]) warn of potential long-term societal risks associated with a legalization of PAS (e.g., *slippery slope*). Opposing statements (AMDA [27], ACP [19], and NHPCO [26]) and 1 studied neutrality statement (APA [24]) suggest that effective palliative care can diminish a patient’s desire for PAS.

## Discussion

This study has several key findings. First, of 150 secular US medical and surgical professional societies, only 12 (8%) have position statements on PAS and euthanasia: 11 for PAS (5 opposing and 4 studied neutrality) and 9 for euthanasia (6 opposing and 2 studied neutrality). Only 3 of these statements have been published in peer-reviewed journals. Second, opposing and studied neutrality statements use similar linguistic sentiment. Third, although opposing and studied neutrality statements have clear differences, they also share recommendations.

It is unclear why so few US societies have position statements on PAS and euthanasia. A given society’s lack of a statement regarding these topics may be due to numerous factors. For example, large and influential societies such as AMA and ACP have position statements on PAS and euthanasia, potentially inhibiting smaller societies from taking positions or causing the smaller societies to perceive their taking a position as unnecessary or irrelevant. Indeed, in personal communications with representatives of societies that do not have position statements, some representatives directed us to the AMA website (J. Barsness, written communication, June 2018). Society specialty may be another factor. For example, in an email from a specialized surgical society, the contact cited the highly specialized field of its members as a reason for not having a position statement (J. Barsness, written communication, June 2018). These phenomena, along with the controversy surrounding the topics, may discourage a society from creating a position statement about PAS or euthanasia (or both).

No position statement argued in favor of PAS or euthanasia. In consideration of a general desire to avoid controversy, a position of studied neutrality may be perceived by a professional society as the only feasible alternative to an opposing position. The decisions of several societies to acknowledge PAS or euthanasia without taking a position appear similar in intent. Either ethical uncertainty exists in the field or the societies believe the need exists to suppress potentially controversial statements.

Among available position statements, linguistic trends are apparent. Societies with studied neutrality positions emphasize patient autonomy regarding end-of-life decision-making but also respect the physician’s role as a health care provider and recognize the benefit of palliative care [16, 24]. The AAN, which takes a studied neutral position, reasons that,

“The Ethics, Law, and Humanities Committee endorses the belief that the primary role of a physician is to prevent and treat disease whenever possible. At the same time, the committee strongly endorses the provision of palliative care to alleviate suffering in patients with illnesses that are unresponsive to disease-specific treatments” [16].

Studied neutrality statements typically call for rigorous ethical training and further study of palliative care and PAS. This call leaves open the possibility of further analysis [17]. The APA, taking a studied neutrality position, reasons, “[We] encourage psychologists to obtain training in ethics (e.g., medical ethics, professional codes of conduct) in the context of diversity, as applied to palliative and end-of-life decisions and care” [24].

Societies with opposing statements view PAS as contrary to the physician’s role in the general US society, do not view death as a right, and view that patient autonomy is an insufficient reason for legalization of PAS. The statements of the AMDA, ACP, AMA, and ASA posit that PAS contradicts the role of a physician [19, 20, 25, 27]. The ACP position statement provides illustrative reasoning: “Physician-assisted suicide requires physicians to breach specific prohibitions as well as the general duties of beneficence and nonmaleficence. Such breaches are viewed as inconsistent with the physician’s role as healer and comforter” [19]. Unsurprisingly, the opposing statements do not mention further study of PAS. Statements that oppose euthanasia follow similar reasoning.

Some opposing statements (of AMDA, ACP, and NHPCO) reference the permissibility of palliative sedation to counter the claim that PAS is a legitimate symptom management approach in the case of extreme discomfort [19, 26, 27]. In such cases, patients should receive aggressive palliation. AMDA reasons, “AMDA supports aggressive treatment toward relieving the pain, anxiety, depression, emotional isolation, and other physical symptoms that can accompany the dying process even if the unintended result of such treatment may hasten the patient’s death” [27]. The ACP and NHPCO statements also highlight the US Supreme Court’s prior ruling that there is no right to die (or PAS) in the United States [19, 26].

Of note, studied neutrality statements (AAHPM and AAN) [13, 16] and opposing statements (ACP and AMA) [19, 20] warn of a slippery slope of long-term risks that PAS legalization may incur. The ACP opposing statement says, “Although the ACP’s fundamental concerns are based on ethical principles, research suggests that a ‘slippery slope’ exists in jurisdictions where physician-assisted suicide and euthanasia are legal” [19]. The AAHPM’s studied neutral statement says, “Such a change risks unintended long-range consequences that may not yet be discernible, including effects on the relationship between medicine and society” [13]. Such long-range consequences include broadened use of PAS for nonterminal conditions and use of PAS in favor of palliative care. Additionally, the studied neutrality and opposing statements suggest that effective palliative care can diminish a patient’s desire for PAD [19, 24, 27, 29]. The AAN’s studied neutrality statement says,

“[The committee] expresses support for improved availability of palliative care services, palliative care education for AAN members, and palliative care research intended to identify more effective means to alleviate refractory suffering of dying patients. By doing so, it hopes to minimize future patient interest in hastened death” [16].

The language of position statements correlates with the position taken by the societies. For instance, studied neutrality statements refrain from the word *suicide* and instead use such terms as *hastened death* or *assisted death* [13, 16, 24]. This use of language is justified in the AAN statement as a means of reducing stigma associated with PAS [16]. Alternative terminology regarding PAS has been actively considered in the literature [30]. In contrast, the ACP opposing statement states a rationalization of use of the word *suicide* as not being derogatory and aiding in clarity in the discussion of the topic (e.g., in contrast to “physician aid in dying,” which could refer to palliative care, terminal sedation, PAS, and euthanasia) [19]. Unsurprisingly, statements that oppose PAS or euthanasia (or both) use the word *suicide* more frequently [19–21, 27, 29]. In contrast, studied neutrality statements use *psychologists*, *law*, and *individuals* more frequently than opposing statements—likely a reflection of the procedures used to request PAS and the emphasis on patient autonomy in association with PAS. That said, the most popular words used in the opposing statements and studied neutrality statements are similar.

Statements have considered the culturally and historically negative connotations of the term *suicide* [19]. Recognizing these connotations, we sought to explore whether the opposing statements’ preference for referring to PAS as a *suicide* and studied neutrality statements’ preference for alternative terms such as *assisted death* indicates a statement’s comprehensive use of emotionally positive or negative language. Therefore, we extended the linguistic analysis by exploring possible associations between linguistic sentiment and statement position through comparison of mean sentiment scores across position categories. Although studied neutrality statements commonly use phrases such as *assisted death* in place of *suicide*, our linguistic analysis showed similar mean sentiment scores for studied neutrality statements and opposing statements. Furthermore, both opposing and studied neutrality statements tended to use emotionally positive language.

Nonetheless, our analysis and findings provide no insight into the rhetorical decision to use the label *suicide* or an alternate. Rather, our findings suggest that use of *suicide* or an alternate term such as *assisted death* does not indicate that a position statement intentionally uses emotionally positive or negative language.

### Limitations

A limitation of the present analysis is the small number of position statements of the organizations. Corpus-based sentiment analysis would provide a better understanding of author intent in the statements because this approach has the ability to consider phrase context more thoroughly [15]. That said, we did not use this type of analysis because the authors of these position statements were unlikely to use techniques such as sarcasm or irony. In addition, we did not include position statements from societies with religious affiliations, some of which have large memberships (e.g., Christian Medical and Dental Associations [31]). Because our study focused on position statements of US professional societies, we intentionally did not include statements from non-US–based societies. Nonetheless, given the variability of perspectives and laws on PAS and euthanasia globally, a study comparing US with non-US statements should be considered.

## Conclusions

Only a dozen secular US medical and surgical professional societies have position statements on PAS and euthanasia, and only 3 of these statements have been published in peer-reviewed journals. The reasons for these small numbers are unclear but may be related to the controversial nature of the topics, the positions of large and influential societies, and the relevancy of the topics for specific specialty societies.

Aside from the use of the words *suicide*, *medicine*, and *treatment* in opposing statements and the words *psychologists*, *law*, and *individuals* in studied neutrality statements, the most popular words used in opposing and studied neutrality position statements are similar. Use of the word *suicide* or *assisted death* does not appear to indicate a statement’s comprehensive use of emotionally positive or negative language.

Opposing statements generally claim PAS contradicts the healing role of the physician and that alternative approaches to symptom control exist for extreme cases (e.g., palliative sedation). Studied neutrality statements highlight patient autonomy in decision-making associated with the dying process. The opposing statements and the studied neutrality statements cite potential long-term societal risks associated with legalization of PAS and suggest that effective palliative care can diminish a patient’s desire for PAS.

## Supplementary information

**Additional file 1. Table S1**: Positions on PAS and Euthanasia of all Identified US Medical and Surgical Societies.

## Data Availability

All data generated or analyzed during this study are included in this published article and its supplementary information files.

## Abbreviations

AAHPM: American Academy of Hospice and Palliative Medicine
AAN: American Academy of Neurology
ACP: American College of Physicians
AMA: American Medical Association
AMDA: Society for Post-Acute and Long-term Care Medicine
APA: American Psychological Association
ASA: American Society of Anesthesiologists
NHPCO: National Hospice and Palliative Care Organization
PAD: Physician-assisted death
PAS: Physician-assisted suicide
