# Supplementary material for: US medical and surgical society position statements on physician-assisted suicide and euthanasia: a review
Source: BMC Med Ethics. 2020 Nov 3;21:111. doi: 10.1186/s12910-020-00556-5 (PMC7640655; doi:10.1186/s12910-020-00556-5)
Supplement: Supplementary file 1 — Additional file 1. Table S1: Positions on PAS and Euthanasia of all Identified US Medical and Surgical Societies. [file 12910_2020_556_MOESM1_ESM.docx]

**Supplemental Table.** Positions on PAS and Euthanasia of all Identified US Medical and Surgical Societies

| **Society** | **Position on PAS** | **Position on Euthanasia** |
| --- | --- | --- |
| Academy of Physicians in Clinical Research | no statement | no statement |
| Aerospace Medical Association | no statement | no statement |
| AMDA—The Society for Post-acute and Long-term Care Medicine | opposed | opposed |
| American Academy of Allergy Asthma & Immunology | no statement | no statement |
| American Academy of Child and Adolescent Psychiatry | no statement | no statement |
| American Academy of Cosmetic Surgery | no statement | no statement |
| American Academy of Dermatology | no statement | no statement |
| American Academy of Emergency Medicine | no statement | no statement |
| American Academy of Facial Plastic and Reconstructive Surgery | no statement | no statement |
| American Academy of Family Medicine | no statement | no statement |
| American Academy of Family Physicians | no statement | no statement |
| American Academy of Hospice and Palliative Medicine | studied neutrality | no statement |
| American Academy of Insurance Medicine | no statement | no statement |
| American Academy of Neurology | studied neutrality | opposed |
| American Academy of Ophthalmology | no statement | no statement |
| American Academy of Orthopaedic Surgeons | no statement | no statement |
| American Academy of Otolaryngic Allergy | no statement | no statement |
| American Academy of Otolaryngology - Head and Neck Surgery | no statement | no statement |
| American Academy of Pain Medicine | no statement | no statement |
| American Academy of Pediatrics | no statement | no statement |
| American Academy of Physical Medicine and Rehabilitation | no statement | no statement |
| American Academy of Psychiatry and the Law | no statement | no statement |
| American Association for Cancer Research | no statement | no statement |
| American Association for Geriatric Psychiatry | no statement | no statement |
| American Association for Hand Surgery | no statement | no statement |
| American Association for the Study of Liver Diseases | no statement | no statement |
| American Association for Thoracic Surgery | no statement | no statement |
| American Association for Vascular Surgery | no statement | no statement |
| American Association of Clinical Endocrinologists | no statement | no statement |
| American Association of Clinical Urologists | no statement | no statement |
| American Association of Gynecologic Laparoscopists | no statement | no statement |
| American Association of Neurological Surgeons | no statement | no statement |
| American Association of Neuromuscular & Electrodiagnostic Medicine | no statement | no statement |
| American Association of Orthopaedic Medicine | no statement | no statement |
| American Association of Plastic Surgeons | no statement | no statement |
| American Association of Public Health Physicians | no statement | no statement |
| American Clinical Neurophysiology Society | no statement | no statement |
| American College of Allergy, Asthma and Immunology | no statement | no statement |
| American College of Cardiology | no statement | no statement |
| American College of Chest Physicians | no statement | no statement |
| American College of Critical Care Medicine | no statement | no statement |
| American College of Emergency Physicians | no statement | no statement |
| American College of Gastroenterology | no statement | no statement |
| American College of Legal Medicine | no statement | no statement |
| American College of Medical Genetics and Genomics | no statement | no statement |
| American College of Medical Quality | no statement | no statement |
| American College of Mohs Surgery | no statement | no statement |
| American College of Nuclear Medicine | no statement | no statement |
| American College of Obstetricians and Gynecologists | acknowledge | acknowledge |
| American College of Occupational and Environmental Medicine | no statement | no statement |
| American College of Pediatricians | no statement | opposed |
| American College of Phlebology | no statement | no statement |
| American College of Physicians | opposed | opposed |
| American College of Preventive Medicine | no statement | no statement |
| American College of Radiation Oncology | no statement | no statement |
| American College of Radiology | no statement | no statement |
| American College of Rheumatology | no statement | no statement |
| American College of Surgeons | no statement | no statement |
| American Contact Dermatitis Society | no statement | no statement |
| American Diabetes Association | no statement | no statement |
| American Gastroenterological Association | no statement | no statement |
| American Geriatrics Society | no statement | no statement |
| American Gynecological & Obstetrical Society | no statement | no statement |
| American Heart Association | no statement | no statement |
| American Hepato-Pancreato-Biliary Association | no statement | no statement |
| American Institute of Ultrasound in Medicine | no statement | no statement |
| American Medical Association | opposed | opposed |
| American Medical Group Association | no statement | no statement |
| American Medical Rehabilitation Providers Association | no statement | no statement |
| American Medical Society for Sports Medicine | no statement | no statement |
| American Neurological Association | no statement | no statement |
| American Orthopaedic Association | no statement | no statement |
| American Orthopaedic Foot and Ankle Society | no statement | no statement |
| American Pain Society | no statement | no statement |
| American Pancreatic Association | no statement | no statement |
| American Pediatric Surgical Association | no statement | no statement |
| American Pharmacists Association | studied neutrality | studied neutrality |
| American Psychiatric Association | acknowledge | opposed |
| American Psychological Association | studied neutrality | studied neutrality |
| American Roentgen Ray Society | no statement | no statement |
| American Society for Aesthetic Plastic Surgery | no statement | no statement |
| American Society for Clinical Pathology | no statement | no statement |
| American Society for Dermatologic Surgery | no statement | no statement |
| American Society for Gastrointestinal Endoscopy | no statement | no statement |
| American Society for Metabolic and Bariatric Surgery | no statement | no statement |
| American Society for Nutrition | no statement | no statement |
| American Society for Radiation Oncology | no statement | no statement |
| American Society for Reproductive Medicine | no statement | no statement |
| American Society for Surgery of the Hand | no statement | no statement |
| American Society of Abdominal Surgeons | no statement | no statement |
| American Society of Addiction Medicine | no statement | no statement |
| American Society of Anesthesiologists | opposed | no statement |
| American Society of Breast Surgeons | no statement | no statement |
| American Society of Cataract and Refractive Surgery | no statement | no statement |
| American Society of Clinical Oncology | no statement | no statement |
| American Society of Clinical Rheumatologists | no statement | no statement |
| American Society of Colon and Rectal Surgeons | no statement | no statement |
| American Society of Dermatopathology | no statement | no statement |
| American Society of Echocardiography | no statement | no statement |
| American Society of General Surgeons | no statement | no statement |
| American Society of Hematology | no statement | no statement |
| American Society of Interventional Pain Physicians | no statement | no statement |
| American Society of Maxillofacial Surgeons | no statement | no statement |
| American Society of Nephrology | no statement | no statement |
| American Society of Neuroradiology | no statement | no statement |
| American Society of Ophthalmic Plastic and Reconstructive Surgery | no statement | no statement |
| American Society of Pediatric Otolaryngology | no statement | no statement |
| American Society of Plastic Surgeons | no statement | no statement |
| American Society of Retina Specialists | no statement | no statement |
| American Society of Transplant Surgeons | no statement | no statement |
| American Surgical Association | no statement | no statement |
| American Thoracic Society | no statement | no statement |
| American Urological Association | no statement | no statement |
| Association of American Physicians and Surgeons | no statement | no statement |
| Association of Medicine and Psychiatry | no statement | no statement |
| Association of Military Surgeons of the United States | no statement | no statement |
| Association of Pulmonary and Critical Care Medicine Program Directors | no statement | no statement |
| Association of University Radiologists | no statement | no statement |
| Asthma and Allergy Foundation of America | no statement | no statement |
| College of American Pathologists | no statement | no statement |
| Congress of Neurological Surgeons | no statement | no statement |
| Contact Lens Association of Ophthalmologists | no statement | no statement |
| Gerontological Society of America | no statement | no statement |
| Heart Failure Society of America | no statement | no statement |
| Heart Rhythm Society | no statement | no statement |
| Infectious Diseases Society of America | no statement | no statement |
| National Association of Medical Examiners | no statement | no statement |
| National Hospice and Palliative Care Organization | opposed | no statement |
| North American Society of Obstetric Medicine | no statement | no statement |
| North American Spine Society | no statement | no statement |
| Obesity Medicine Association | no statement | no statement |
| Radiological Society of North America | no statement | no statement |
| Renal Physicians Association | no statement | no statement |
| Society for Cardiovascular Angiography and Interventions | no statement | no statement |
| Society for Investigative Dermatology | no statement | no statement |
| Society for Vascular Medicine | no statement | no statement |
| Society for Vascular Surgery | no statement | no statement |
| Society of American Gastrointestinal and Endoscopic Surgeons | no statement | no statement |
| Society of Critical Care Medicine | no statement | no statement |
| Society of General Internal Medicine | no statement | no statement |
| Society of Hospital Medicine | no statement | no statement |
| Society of Interventional Radiology | no statement | no statement |
| Society of Laparoendoscopic Surgeons | no statement | no statement |
| Society of Nuclear Medicine and Molecular Imaging | no statement | no statement |
| Society of Thoracic Surgeons | no statement | no statement |
| Spine Intervention Society | no statement | no statement |
| The Endocrine Society | no statement | no statement |
| The Triological Society | no statement | no statement |
| Undersea and Hyperbaric Medical Society | no statement | no statement |
| United States and Canadian Academy of Pathology | no statement | no statement |
